# Commentary: Sleep Deprivation Promotes Habitual Control over Goal-Directed Control: Behavioral and Neuroimaging Evidence

**DOI:** 10.3389/fnbeh.2018.00082

**Published:** 2018-05-01

**Authors:** Yannick Boddez, Eike K. Buabang, Ann-Kathrin Zenses, Mathilde Descheemaeker

**Affiliations:** ^1^Department of Clinical Psychology and Experimental Psychopathology, University of Groningen, Groningen, Netherlands; ^2^Centre for the Psychology of Learning and Experimental Psychopathology, KU Leuven, Leuven, Belgium; ^3^Research Group of Quantitative Psychology and Individual Differences, KU Leuven, Leuven, Belgium; ^4^Center for Social and Cultural Psychology, KU Leuven, Leuven, Belgium

**Keywords:** conditioning, learning and memory, habits, goal-directed behavior, sleep

Both in layman's and scientific terms, it is common to speak of goal-directed and habitual behavior. In case of goal-directed behavior, the response (e.g., reaching into the cookie jar) is caused by the mental representation of its outcome (i.e., the goal; e.g., chocolate consumption). When behavior is habitual, the response is caused by the situation (e.g., being around the cookie jar).

Chen et al. (2017) hypothesized that sleep loss tips the balance from goal-directed to habitual control, which could explain the association between sleep loss and suboptimal behavior (e.g., overeating). They therefore manipulated whether or not participants were deprived of sleep before the training and the test phase (Experiment 1) or the test phase only (Experiment 2) of a task that models this balance.

During training, participants saw a box with a picture of a piece of fruit on it (stimulus). Participants could press left or right to open the box (response). If they responded correctly, they saw another piece of fruit inside the box (outcome) and earned points. The training phase consisted of three trial types: standard, congruent, and incongruent trials. A description can be found in the target article, but discussion of the incongruent trials is warranted. Incongruent trials consisted of (a) trials in which a type of fruit (stimulus) resulted in another type of fruit (outcome) if a certain response was made (e.g., apple results in banana if one presses right) and (b) trials in which the types of fruit used as stimulus and outcome were reversed and which required the opposite response (e.g., banana results in apple if one presses left). Incongruent trials are supposed to create conflict, because the response caused by the mental representation of a fruit as stimulus (e.g., pressing right caused by the apple as stimulus) is different from the response caused by the mental representation of the same fruit as outcome (e.g., pressing left caused by the apple as outcome). The assumption is that this conflict is solved by regressing to habitual control, as the conflict would dissipate if the mental representation of the outcome no longer caused responding.

However, De Houwer et al. (2018) criticized this assumption and showed that responding on the different trial types, including the incongruent trials, is caused by the representation of an overlooked outcome, namely of earning points (rather than of the fruit outcome or of no outcome at all—as intended in the task).This criticism puts the findings of Chen et al. in a new perspective. An accidental feature of the task is that participants can successfully navigate through the training phase without taking the fruit outcomes into account (Figure 1). That is, they can learn, given a certain fruit stimulus, which response results in earning points without paying attention to which fruit stimulus results in which fruit outcome. If we assume that participants in the sleep deprivation group encode these—in this phase redundant—relations between fruit stimuli and fruit outcomes to a lesser extent than participants in the sleep group, we can explain the results of Chen et al. without relying on habitual control. This assumption is in line with previous findings that sleep deprivation results in impaired encoding (Yoo et al., 2007; Kaida et al., 2015).

**Figure 1:**
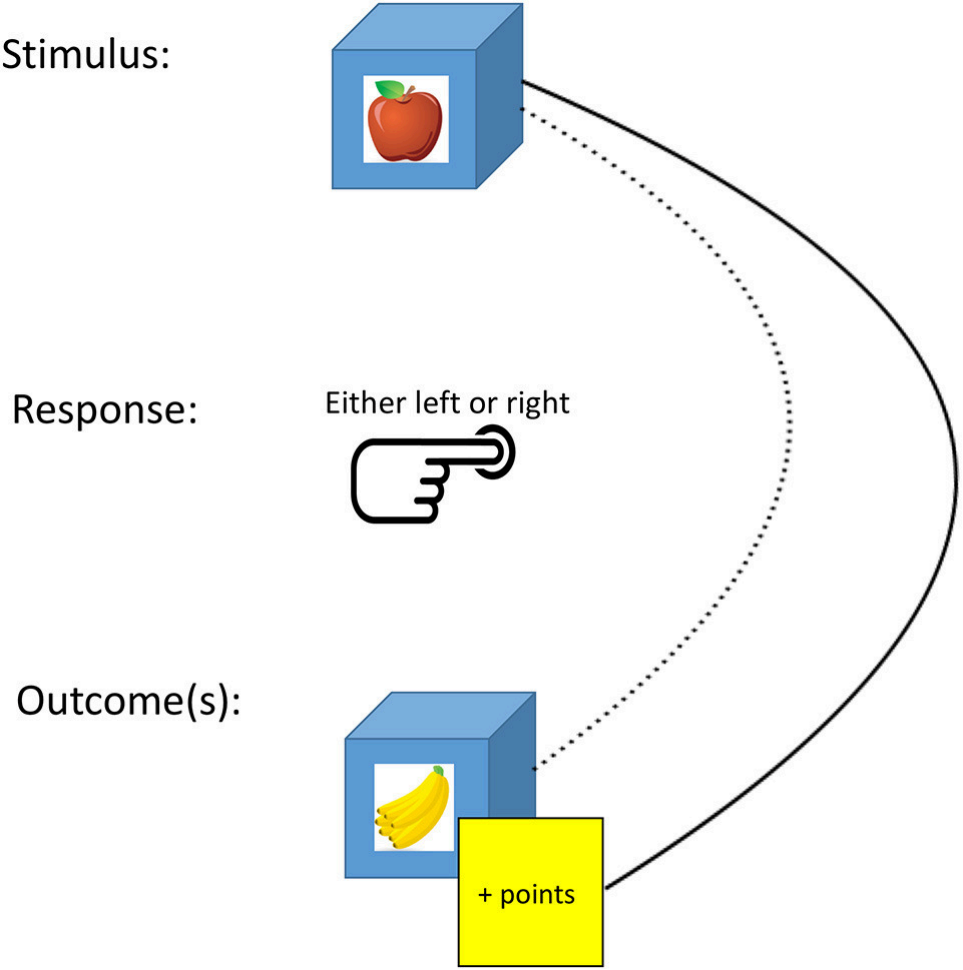
Participants can navigate through the training phase by learning the relations between fruit stimuli, the correct response and earning points (full curved line) and without learning the redundant relations between fruit stimuli, the correct response and fruit outcomes (dotted curved line).

We will now first consider the results of the slips-of-action test, which Chen et al. describe as the critical test of the balance between habitual and goal-directed control. During this test, participants were informed that certain fruit outcomes would no longer result in points and asked to no longer respond when presented with fruit stimuli that were related to these fruit outcomes. In Experiment 1, sleep deprivation resulted in a higher level of uncalled-for responding to these fruit stimuli. According to our alternative explanation, these action slips are no indication of habitual behavior: Participants might entertain the goal of earning points, but make mistakes because they never properly encoded which fruit stimuli are associated with the devalued fruit outcomes. This also allows to explain why Chen et al. did not find an effect of sleep deprivation on the slips-of-action test in Experiment 2, in which the sleep manipulation took place right before the slips-of-action test instead of before the training phase. In such case, sleep deprivation cannot affect the encoding of the relations between fruit stimuli and fruit outcomes and, hence, no between-group differences should be expected. In line with the above, sleep-deprived participants in Experiment 1 had less explicit knowledge about which fruit stimuli resulted in which fruit outcomes than participants in the sleep group, whereas no such difference was found in Experiment 2.

In Experiment 1, Chen et al. also compared brain activity between the sleep deprivation and the sleep control group during the training phase. Their analyses were based on the assumption that incongruent trials involve only habit learning, while the other trials (i.e., standard and congruent trials) involve both goal-directed and habit learning. When contrasting these trial types, there was evidence for less vmPFC activity in the sleep-deprived group. From this, Chen et al. conclude that sleep deprivation leads to less vmPFC activity during trials that require goal-directed learning, which they use to explain the increase in action slips. However, reduced vmPFC activity during the training phase could also merely indicate that sleep-deprived participants are encoding less information about the relationship between fruit stimuli and fruit outcomes, which is in line with evidence that vmPFC activity is positively associated with encoding information about pairs of overlapping stimuli (Preston and Eichenbaum, 2013). The reduced activity in the caudate in sleep-deprived participants during incongruent trials can be understood in the same way, since the caudate is known to be active during encoding of reward learning (Seger and Cincotta, 2005).

In conclusion, we present an alternative explanation for the findings of Chen et al. Our commentary sparks debate about what is needed to substantiate that behavior is in fact habitual (Moors et al., 2017). Watson and de Wit (2018) discuss limitations of current paradigms and suggest avenues for future research.

## Author contributions

YB, EB, A-KZ, and MD discussed the paper by Chen et al. over the course of several meetings. YB wrote the paper with critical additions and revisions by EB, AZ, and MD.

### Conflict of interest statement

The authors declare that the research was conducted in the absence of any commercial or financial relationships that could be construed as a potential conflict of interest.
